# ChIP-Array 2: integrating multiple omics data to construct gene regulatory networks

**DOI:** 10.1093/nar/gkv398

**Published:** 2015-04-27

**Authors:** Panwen Wang, Jing Qin, Yiming Qin, Yun Zhu, Lily Yan Wang, Mulin Jun Li, Michael Q. Zhang, Junwen Wang

**Affiliations:** 1 Centre for Genomic Sciences and Department of Biochemistry, LKS Faculty of Medicine, The University of Hong Kong, Hong Kong SAR, China; 2 Shenzhen Institute of Research and Innovation, The University of Hong Kong, Shenzhen, Guangdong 518057, China; 3 Bioinformatics Division, TNLIST, Tsinghua University, Beijing 100084, China; 4 Department of Molecular and Cell Biology, Center for Systems Biology, The University of Texas at Dallas, Dallas, TX 75080, USA

## Abstract

Transcription factors (TFs) play an important role in gene regulation. The interconnections among TFs, chromatin interactions, epigenetic marks and cis-regulatory elements form a complex gene transcription apparatus. Our previous work, ChIP-Array, combined TF binding and transcriptome data to construct gene regulatory networks (GRNs). Here we present an enhanced version, ChIP-Array 2, to integrate additional types of omics data including long-range chromatin interaction, open chromatin region and histone modification data to dissect more comprehensive GRNs involving diverse regulatory components. Moreover, we substantially extended our motif database for human, mouse, rat, fruit fly, worm, yeast and *Arabidopsis*, and curated large amount of omics data for users to select as input or backend support. With ChIP-Array 2, we compiled a library containing regulatory networks of 18 TFs/chromatin modifiers in mouse embryonic stem cell (mESC). The web server and the mESC library are publicly free and accessible athttp://jjwanglab.org/chip-array.

## INTRODUCTION

Deciphering gene regulatory network (GRN) is crucial to understanding the mechanisms of various biological processes and the onset of diseases. Gene transcription can be regulated by multiple factors, such as transcription regulation, epigenetics and post-translational modifications. Previous version of ChIP-Array incorporates *in vivo* transcription factor (TF) binding and transcriptome data to construct a GRN for a TF (1). The TF's bindings are detected by chromatin immunoprecipitation followed by high-throughput technologies (ChIP-seq/chip/exo, collectively, ChIP-X), and the transcriptome data are obtained before and after the TF's perturbation, by either microarray or RNA-sequencing. However, TFs often collaborate with other factors for gene regulation. Recent studies showed that interplay among different TFs, chromatin modifiers and/or epigenetic marks comprehends the functional diversity of gene regulation (2–5). For example, super enhancers aggregating multiple TFs, transcription cofactors, chromatin regulators and polymerase are found to be associated with highly expressed genes that control cell identity and disease (6). Some enhancers can locate as far as 100 kb away from their target genes through chromatin loops, which is hard to identify with traditional technology. Fortunately, the development of advanced chromatin conformation capturing technologies, such as Hi-C and Chromatin Interaction Analysis by Paired-End Tag sequencing (ChIA-PET), enables us to detect long-range enhancers with less false positives (7). Thus, the omics data quantifying these chromatin events provide valuable information to improve GRN construction. Other information, for instance, genome-wide open chromatin regions are also helpful to improve the quality of TF-target identification, since TF binding can be impeded and dissociated by nucleosome (8), and most of TF binding sites (TFBSs) identified by the Encyclopedia of DNA Elements (ENCODE) consortium are located in high DNA-accessible regions (9).

In spite of the development of high-throughput technologies and the emergence of multiple omics data conducted on different regulatory factors, existing tools have not sufficiently integrated those data for GRN construction. Most of these tools either combine only ChIP-X and transcriptome data to reduce the false-positive rate (2,10–12), or require a large number of samples to build their models for the identification of interactions among different types of factors, such as genetic variation, DNA methylation and miRNA (5,13,14) (Supplementary Section 4). Hence, we developed our enhanced version of ChIP-Array, which is able to incorporate not only ChIP-X and transcriptome data, but also long-range chromatin interaction, open chromatin region and histone modification data, to construct GRNs.

The new version can also construct regulatory networks triggered by a cellular event, such as a change in histone modification, or a cellular perturbation (knock in, knock out, drug treatment, or pathogen infection). To take advantages of public omics data, we also manually curated large volume of omics data of various cell types/lines for users to use in combination with their own data. The algorithm for target identification is also enhanced. In addition to directly determining the targets as the intersection of TFBS-enriched and differentially expressed genes (DEGs) in previous version, we offer another target detection method, ‘Rank Product’, which is based on relative peak position to transcription start site (TSS), peak intensity and gene expression change (2,15). Besides, we substantially extended our TF motif database to 6584 position weight matrices (PWMs) of 4727 TFs, and for more species including human, mouse, rat, fruit fly, worm, yeast and *Arabidopsis*. To provide better user experience, the web interface is more user-friendly. The events, including TF-binding, long-range chromatin interaction, open chromatin region and histone modification, around a TSS, are intuitively displayed in JBrowse (16). Furthermore, enriched Gene Ontology (GO) and Pathways from the resulting GRN are displayed in the result page. With this new web server, we constructed a network library containing regulatory networks of 18 TFs/chromatin modifiers in mouse embryonic stem cell (mESC) and made it freely accessible.

## DESCRIPTION OF ChIP-ARRAY 2

### Input data

The main input data contain three parts (Supplementary Table S2): (i) a list of genomic positions containing TF/chromatin modifier's binding peak regions, differential histone modification regions or differential DNA methylation regions. The centered regulator in the constructed GRN can be a TF, a type of histone modification or a biological perturbation, respectively; (ii) a list of differentially expressed genes under the perturbation of a TF/chromatin modifier, or other treatment; (iii) other types of omics data such as long-range chromatin interactions, Dnase hypersensitive sites (DHS) and various histone modifications. For TF binding data, users can either upload their own data, or use our curated ChIP-X data or TFBSs predicted by PWMs (17,18). Besides the general format ‘BED’ and ‘GFF’, the server supports peak files generated by popular peak calling tools including CisGenome (19) and MACS (20). To take into account the biological replicates, users can use the peak file generated by PePr (21). To consider differential TF binding or histone modification between two experimental conditions, users can use peak file generated by DBChIP (22) and DiffBind (23). For transcriptome data, ChIP-Array 2 is able to read the standard output of LIMMA (24) and Cuffdiff (25). The server also supports a simple format (gene ID, log_2_(fold change) and statistical value) for the essential information of gene expression changes. For convenience, in the transcriptome profile, most commonly-used array probe names and various gene IDs from different platforms and sources can be recognized by ChIP-Array 2. Users can either upload other types of omics data in the ‘BED’ format or select our curated data from the web server (Supplementary Section 2.1). They can also select the information of enhancers from VISTA database (26) and other experimentally-validated enhancers we curated from literature (Supplementary Section 2.2) for target detection. A sample result and a copy of sample data are available for users to browse before they run their own jobs.

### Workflow

**Figure 1.**
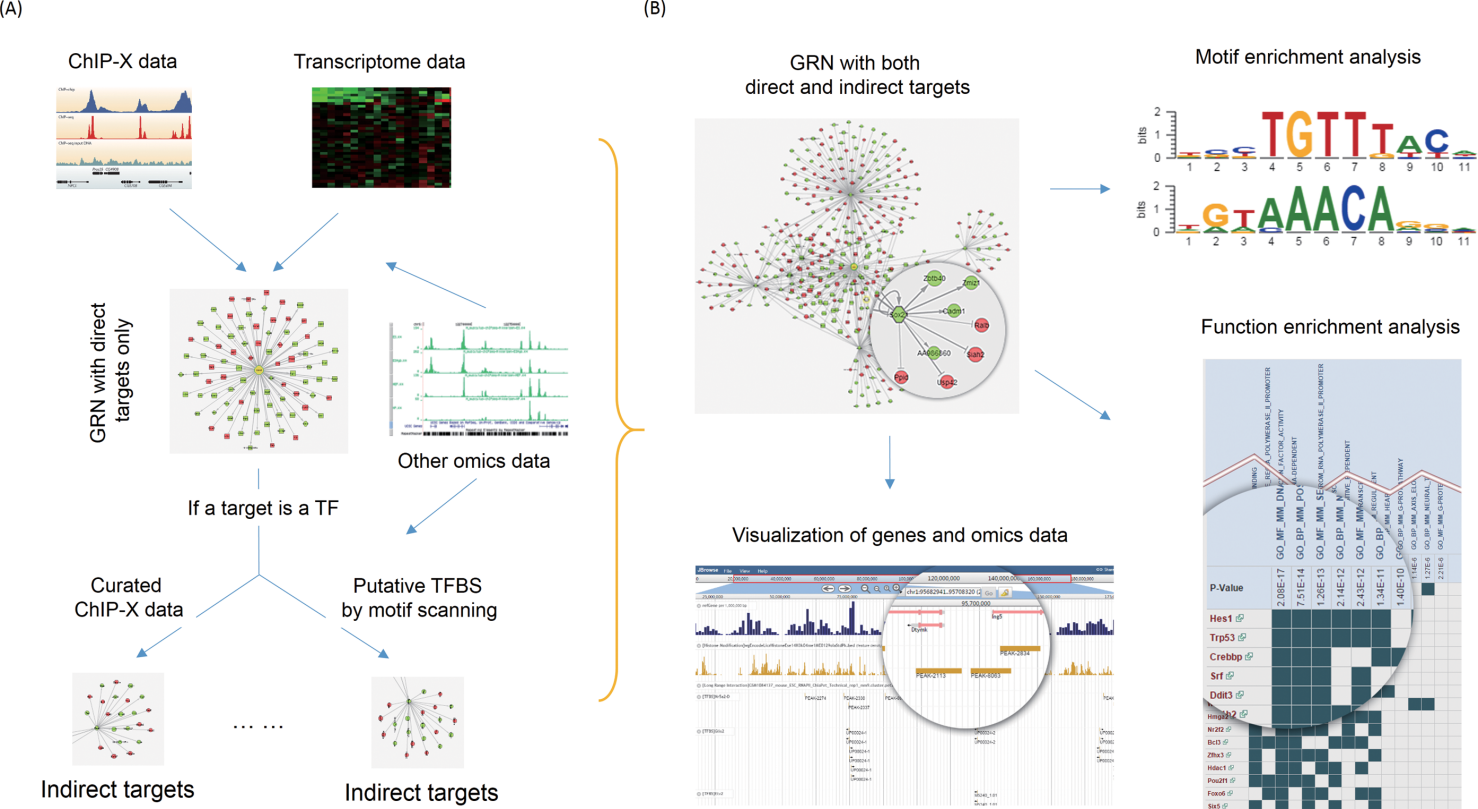
Workflow (A) and results (B) of ChIP-Array 2. (**A**) Direct targets are identified by combining ChIP-X and transcriptome data. Interplays between the TF of interest and other regulatory factors/target genes are supported by other omics data. Then indirect targets are detected by curated ChIP-X data or predicted TFBSs with the assistance of other omics data. (**B**) The results are composed of four parts: the resulting GRN shown in CytoscapeWeb, motif enrichment analysis by MEME Suite, functional enrichment analysis, and visualization in JBrowse.

The main workflow of ChIP-Array 2 is shown in Figure 1A, ChIP-X and transcriptome data will be combined to detect direct targets, using either the ‘Direct’, by which the targets will be determined as intersection of ChIP peak-enriched genes and DEGs, or ‘Rank Product’ method (Supplementary Section 1.2). In the later method, genes are ranked by both peak abundance and expression changes, and scored by the rank product. When a cutoff is used, genes with higher peak abundance and higher expression change are chosen. Here higher peak abundance denotes more peaks with higher intensity and shorter distance to TSS. To detect TF-target relation involved in enhancer-promoter interaction, long-range chromatin interaction data is used to incorporate the TSSs with peaks located in the distal interacting regions. If the direct target is a TF, the same method will be applied to detect its downstream targets with our curated ChIP-X data, or putative TFBSs predicted by Position Weigh Matrix (PWM) method (Supplementary Section 1.1). When putative TFBSs are used, open chromatin regions and histone modifications are further used to filter out the inactive, non-tissue-specific TFBSs. Finally, the results will be presented in a web page, and all the intermediate and resultant files are downloadable for users to perform further analysis. Complement to single factor analysis, we offer an opportunity for users to study the synergy among regulatory factors by co-occupancy analysis (Supplementary Section 1.3).

### Result page

As shown in Figure 1B, the result page consists of four main parts. The first part is a GRN shown in CytoscapeWeb (27) or rendered by HTML5 using cytoscape.js (http://js.cytoscape.org/) if flash is not supported by user's browser. By default, the number of nodes is limited to about 80 to avoid heavy burden of displaying the network. However, users can choose to show the full network by clicking the link on the right side. Each node or edge is clickable to show the details including the regulation relations, open chromatin regions, histone modifications and binding regions. These regions can be highlighted in next tab in JBrowse. If the TFBSs are detected by ChIP-X data, the motif enrichment analysis will be performed by MEME Suite (28), which can discover *de novo* motifs enriched in the surrounding region. MEME Suite will also compare the discovered motifs and the known motifs in our database, and find the potential co-occupied factors for the query factor. The server can also perform gene set enrichment analysis for the network inferred, against Gene Ontology (GO) terms and annotated pathways. A gene/gene-set overlap matrix is generated accordingly, in which a gene is highlighted if it is present in a particular GO term/pathway. For humans, the gene sets of GO and pathways are downloaded from MSigDB (29). The gene sets for other species are obtained from http://bioinformatics.sdstate.edu/gskb/db/, generated by bioinformatics research group of South Dakota State University.

## EXAMPLES

**Figure 2.**
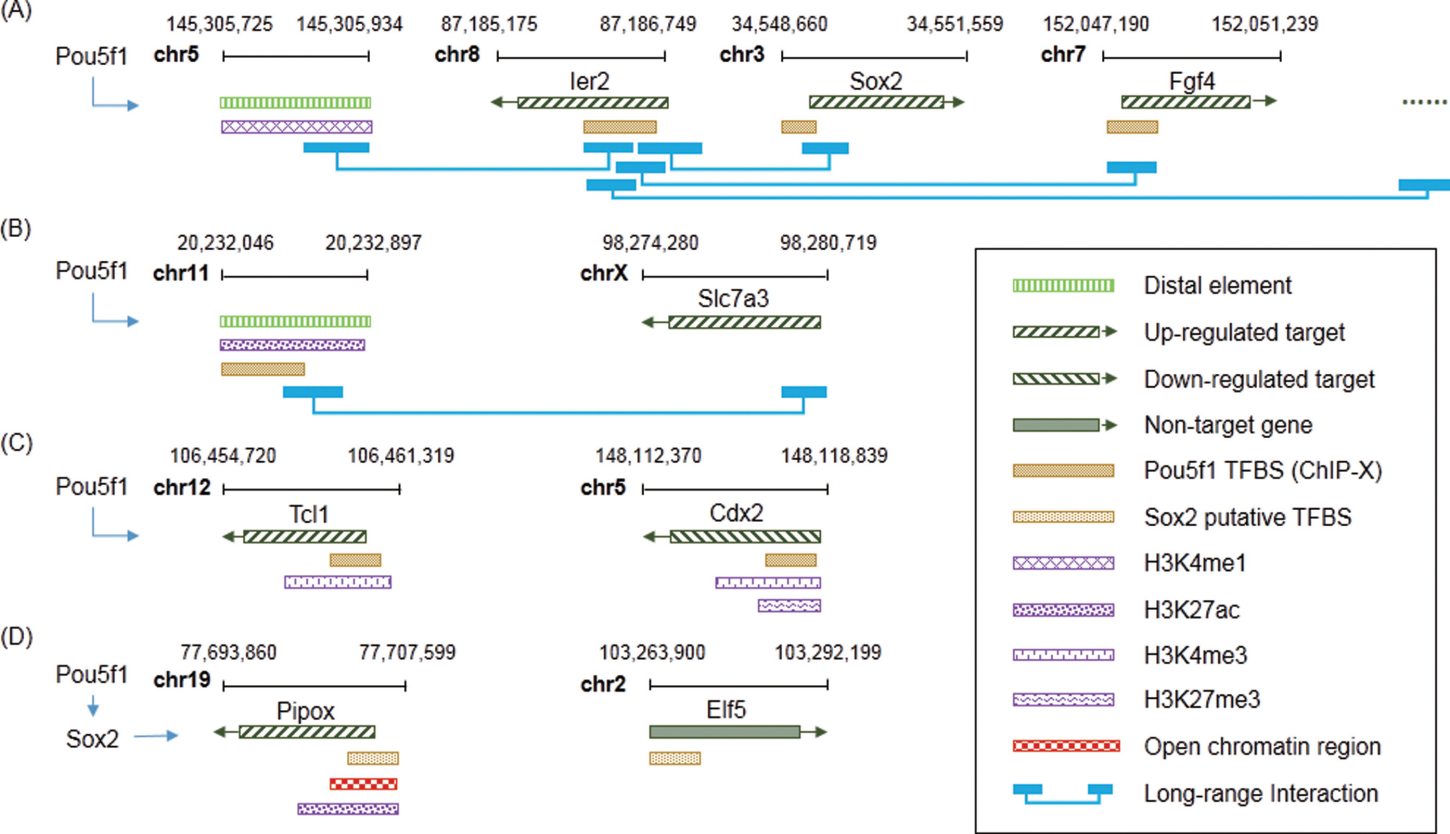
Example targets of Pou5f1 identified by ChIP-Array 2 with multiple omics data. (**A**) A group of genes and distal elements connect to each other through long-range chromatin interactions. Each of the genes has the Pou5f1 binding signals from ChIP-seq, and knockdown of Pou5f1 significantly changes the gene expression. (**B**) Distal elements containing ChIP-seq peaks or putative TFBSs may link to the target genes without significant binding sites. These targets may be overlooked by previous tools where the long-range chromatin interactions are not considered. (**C**) Target genes can be activated (Tcl1) or suppressed (Cdx2) by Pou5f1 when the histone modifications at their promoters are different. (**D**) Histone modification marks and open chromatin regions are used to filter the putative TFBSs for indirect target identification. Since they indicate the tissue-specific accessibility of the TFBSs, TFBSs located in these regions are more likely to be active than those beyond the regions. Thus, putative TFBSs outside of histone modification marks and open chromatin regions are not considered for indirect target inference.

With the omics data we collected from Gene Expression Omnibus (GEO) (Supplementary Table S3), we built a TF/chromatin modifier GRN library for mESC. It contains GRNs controlled by 18 TFs/chromatin modifiers. This library combines data from ChIP-seq of the TFs/chromatin modifiers, transcriptome profiling under their perturbations, together with ChIA-PET, DNase-seq and ChIP-seq of H3K4me3, H3K4me1, H3K27ac, H3K27me3 and H3K9me3. GRNs can be viewed in ‘mESC Library’. Integration of diverse omics data significantly improves the target detection and our understanding of gene transcription regulation (Figure 2 and Supplementary Section 4). For example, with ChIA-PET data, ChIP-Array 2 is able to find gene clusters that are highly related to the functions of the TF of interest (Figure 2A). Pou5f1 (also known as Oct4, an important TF for the pluripotency of mESC), which binds on the promoters of Sox2 and Fgf4, is well known to regulate these two genes (30,31). Through long-range interaction, we found that both genes are connected to Ier2, whose promoter is also bound by Pou5f1. Ier2 is a member of immediate early response proteins that can be induced by the cytokine leukaemia inhibitory factor (LIF), and consistently shows significant down-regulation under the knockdown of Pou5f1 in mESC (32–34). Results from ChIP-Array 2 indicate the co-regulation of Ier2, Sox2 and Fgf4 by Pou5f1 via the aggregating evidence of Pou5f1 binding, down-regulation under Pou5f1 knockdown and the physical interactions among their promoters, which has not been reported by individual studies generating these omics data. Besides, long-range chromatin interaction links TF binding sites in distal elements to the target gene promoter.

ChIP-Array 2 also shows target genes that do not have significant TF binding sites in promoters, but are physically linked to a distal element with a strong TF binding signal (Figure 2B). For instance, although previous studies have reported high correlation between the expressions of Pou5f1 and Slc7a3 (5,32), the mechanism on how they are regulated was not clear. ChIP-Array 2 provides the direct evidence on how Pou5f1 regulates Slc7a3 through an inter-chromosomal interaction between an enhancer and the Slc7a3 promoter. These targets may be overlooked by previous methods in which long-range chromatin interactions were not considered. In addition, ChIP-Array 2 allows the study of interplays between TF and histone modifications (Figure 2C). Tcl1 and Cdx2 are two targets activated and repressed by Pou5f1, respectively. Promoters of both genes are bound by Pou5f1, however, Tcl1 promoter is modified with H3K4me3 but not H3K27me3, while Cdx2 promoter is bivalently modified by both marks in mESC. This implies that different histone modifications at Pou5f1 binding promoters may act differently, causing up- or down-regulation effects on the targets. Moreover, incorporating these histone modifications, as well as data identifying open chromatin regions, we successfully filtered inactive putative TFBSs predicted by sequence scanning when detecting indirect targets. Figure 2D shows two example genes. When searching indirect targets of Pou5f1, both Pipox and Elf5 contain predicted Sox2 binding sites on their promoters. They are significantly down- and up-regulated respectively when Pou5f1 is knocked down. Since Sox2 is a direct target of Pou5f1, both Pipox and Elf5 are identified as indirect targets of Pou5f1 by the old version of ChIP-Array. However, combining the histone modifications and open chromatin regions via ChIP-Array 2, which mark the chromatin accessibility of TFBSs, we found that only Pipox is the true target of Sox2. Elf5 is filtered out because of lacking any marks to support the activity of the predicted Sox2 binding site on its promoter. The direct regulation between Sox2 and Pipox is also confirmed by a ChIP-seq peak of Sox2 on its promoter and expression change when Sox2 is knocked down, while Elf5 shows no evidence in these studies (data not shown) (32,35,36). All these findings can be visualized via JBrowse in the Pou5f1 network stored in ChIP-Array 2 mESC library by inputting the genomic positions of these genes and ticking the relevant data tracks in the full-screen mode of JBrowse.

## DISCUSSION

ChIP-Array 2 integrates ChIP-X and transcriptome data, together with other omics data to construct GRNs. However, it does not require all the above data, and can run even without either ChIP-X or transcriptome data. If transcriptome data are not provided, only direct targets will be detected by ChIP-X data or predicted TFBSs, but no indirect targets will be inferred due to the high false-positive rate. Without ChIP-X data for direct target detection, TFBSs obtained by computational prediction will be utilized to infer the putative targets. To reduce the false positives, users are encouraged to use at least one type of omics data to detect active binding sites for the query factor. Unlike a standalone program, which may run hours for a job, ChIP-Array 2 aims to produce the results in tens of minutes, or even minutes. Some of the parameters are set in advance to reduce running time, but the flexibility may be lost accordingly. For instance, the putative TFBSs are scanned at several certain cutoffs, and then stored on the server, so that users can only select the cutoffs that we used in the pre-scanning. For the motif analysis, we just use peaks in promoters of targets, ignoring other peaks since it may take up to hours for motif discovery. Users can download the job and extract all the peaks and perform the whole-genome motif analysis using standalone MEME Suite.

## SUPPLEMENTARY DATA

Supplementary Data are available at NAR Online.

SUPPLEMENTARY DATA
