# Supplementary material for: ChIP-Array 2: integrating multiple omics data to construct gene regulatory networks
Source: Nucleic Acids Res. 2015 Apr 27;43(Web Server issue):W264–9. doi: 10.1093/nar/gkv398 (PMC4489297; doi:10.1093/nar/gkv398)
Supplement: SUPPLEMENTARY DATA [file supp_43_W1_W264__index.html]

ChIP-Array 2: integrating multiple omics data to construct gene regulatory networks — ChIP-Array 2: integrating multiple omics data to construct gene regulatory networks — ChIP-Array 2: integrating multiple omics data to construct gene regulatory networks — SUPPLEMENTARY DATA 

# ChIP-Array 2: integrating multiple omics data to construct gene regulatory networks

## SUPPLEMENTARY DATA

**Files in this Data Supplement:**

- SUPPLEMENTARY DATA
