# Supplementary material for: ChIP-Array 2: integrating multiple omics data to construct gene regulatory networks
Source: Nucleic Acids Res. 2015 Apr 27;43(Web Server issue):W264–9. doi: 10.1093/nar/gkv398 (PMC4489297; doi:10.1093/nar/gkv398)
Supplement: SUPPLEMENTARY DATA [file supp_gkv398_nar-00443-web-b-2015-File004.docx]

**Supplementary Information**

ChIP-Array 2: integrating multiple omics data to construct gene regulatory networks

Panwen Wang^1,2,#^ , Jing Qin^1,2,#^, Yiming Qin^1^, Yun Zhu^1,2^, Lily Yan Wang^1,2^, Mulin Jun Li^1,2^, Michael Q. Zhang^3,4^, Junwen Wang^1,2,*^

^1^Centre for Genomic Sciences and Department of Biochemistry, LKS Faculty of Medicine, The University of Hong Kong, Hong Kong SAR, China; ^2^Shenzhen Institute of Research and Innovation, The University of Hong Kong, Shenzhen, Guangdong 518057, China; ^3^Bioinformatics Division, TNLIST, Tsinghua University, Beijing 100084, China and ^4^Department of Molecular and Cell Biology, Center for Systems Biology, The University of Texas at Dallas, Dallas, TX 75080, USA

^#^ The authors wish it to be known that, in their opinion, the first two authors should be regarded as joint first authors.

* To whom correspondence should be addressed. Tel: +852 2831 5075; Fax: +852 2855 1254; Email: [junwen@](mailto:junwen@uw.edu)uw.edu

Contents

[ChIP-Array 2: integrating multiple omics data to construct gene regulatory networks 1](#_Toc416280825)

[1 Methods 3](#_Toc416280826)

[1.1 Motif scanning 3](#_Toc416280827)

[1.2 Target detection method 3](#_Toc416280828)

[1.3 Co-occupancy analysis 4](#_Toc416280829)

[1.4 Biological scenario which ChIP-Array 2 can be applied to 4](#_Toc416280830)

[2 Curated Data 6](#_Toc416280831)

[2.1 ChIP-X, long-range interaction data, open chromatin regions and histone modification data 6](#_Toc416280832)

[2.2 Experimental-validated enhancers 6](#_Toc416280833)

[3 mESC library 6](#_Toc416280834)

[4 Comparison with ChIP-Array and other tools 8](#_Toc416280835)

[4.1 Comparison with BETA and ChIP-Array 8](#_Toc416280836)

[4.2 Comparison with other tools 9](#_Toc416280837)

[Reference 11](#_Toc416280838)

# 1 Methods

1. Motif scanning

If ChIP-X data are not available, users can choose to use predicted putative TFBSs by motif scanning in the conserved regions around TSSs (±100kb) for direct or indirect target inference. These TFBSs are scanned and stored on the server in advance. The conservation is measured by the phastCons ([1](#_ENREF_1)) scores, which are downloaded from UCSC ([2](#_ENREF_2)) for all the species supported in ChIP-Array 2 except *Arabidopsis*. The conservation score of *Arabidopsis* is curated from ([3](#_ENREF_3),[4](#_ENREF_4)) . The P-value is calculated based on a null distribution of conservation scores, which are generated by randomly-picked sequences in intergenic regions. The motifs, presented in position weight matrices (PWMs), in our database are extended from 1,151 PWMs of 894 TFs to 6,584 PWMs of 4,727 TFs (Supplementary Table S1). The number of supported species is also increased from 5 to 7. MISP (<http://www.bytebucket.org/hanfeisun/misp>), which implements the algorithm from MOODS ([5](#_ENREF_5)), is used to perform the motif scanning.

Supplementary Table S1. New collection of PWMs in 7 species.

|  | Database | human | mouse | rat | fruit fly | worm | yeast | *Arabidopsis* |
| --- | --- | --- | --- | --- | --- | --- | --- | --- |
| No. of PWMs | CIS-BP | 2995 | 2881 | 2771 | 2123 | 1344 | 788 | 414 |
|  | hPDI | 437 | 437 | 437 | - | - | - | - |
|  | jaspar | 205 | 205 | 205 | 131 | 15 | 177 | 64 |
|  | UniPROBE | 450 | 450 | 450 | 16 | 49 | 163 | 6 |
|  | MacIsaac et al. | - | - | - | - | - | 124 | - |
|  | SCPD | - | - | - | - | - | 23 | - |
|  | YeTFaSCo | - | - | - | - | - | 244 | - |
| No. of TFs | | 1246 | 1144 | 984 | 374 | 159 | 232 | 588 |

1. Target detection method

In our previous version, we used a direct way to detect target genes as the intersection set of TFBS-enriched genes and DEGs, which assigns equal weight for each target. Additionally in this new version, we offered another method called “Rank Product”, based on the rank of the peak concentration and expression changes. The peak concentration is defined as follow ([6](#_ENREF_6)) ([7](#_ENREF_7)):

$PC=\sum_{i=1}^{n} p_{i}e^{-(0.5+4d_{i}/d_{0})}$,
where *d_0_* is a constant distance (i.e. 10kb) around the TSS. It defines a region where the peaks are considered to have contribution to the binding. *d_i_* is the distance of the *i*th peak to the TSS in this region. When this distance increased, the contribution of this peak to the peak abundance is exponentially decreased. *p_i_* is the intensity of the peak. If this value is not offered in input binding information, it will be set to 1; and the method will fall back to the one used in BETA ([8](#_ENREF_8)). Suppose there are *n* significantly differentially-expressed genes with *PC* > 0, which will have two ranks: rank by peak abundance in descending order (*R_pc_*) and false discovery rate (FDR)/P-value from the expression profile in ascending order (*R_de_*). The rank product of each gene, *RP* = (*R_pc_/n*)*(*R_de_/n*), can be regarded as a P-value denoting the probability a gene with peak concentration ranks higher than *R_pc_* and expression change ranks higher than *R_de_* ([8](#_ENREF_8),[9](#_ENREF_9)). With a given cutoff, users are more likely to select the true targets. If the cutoff is set to 1, then the method becomes “direct”, that is, the target gene set is the intersection of TFBS-enriched genes and DEGs.

To detect TF-target relation involved in enhancer-promoter interaction, long-range interaction data are applied when calculating the peak abundance. The peaks will be mapped to the interacting regions, and those mapped peaks will also contribute to the peak abundance hence affect the target ranking. If the TFBSs by motif scanning are used instead of ChIP-X data, open chromatin region and histone modification data will be used to pick the peaks located in the open chromatin regions and demanded histone modification regions.

1. Co-occupancy analysis

Gene transcription initiation and regulation is a complex process, especially in eukaryotes. It involves various proteins binding and working together at the regulatory DNA regions. Complement to single factor analysis, we offer an opportunity for users to study the synergy among regulatory factors by co-occupancy analysis. Users may want to check the co-targets of multiple factors and further to investigate the synergy of these factors. They can merge the GRNs generated by each single factor. Consider the consistence, only the GRNs constructed under the same genome assembly can be merged. In JBrowse, all the tracks from the single job will be remained, and functional enrichment analysis will be performed on all the combined targets.

1. Biological scenario which ChIP-Array 2 can be applied to

Supplementary Table S2. Biological scenario that ChIP-Array 2 can be applied to

| Experiment design | Factor of interest | Part 1 | Part 2 | Part 3 |
| --- | --- | --- | --- | --- |
| Targets of a TF in a certain tissue/cell line | TF | ChIP-X of the TF in the tissue/cell line | Transcriptome before and after (overexpression or knockdown/out) of the TF | Other omics data in the same tissue/cell line |
| Targets of a chromatin modifier in a certain tissue/cell line | Chromatin modifier | ChIP-X of the chromatin modifier in the tissue/cell line | Transcriptome before and after (overexpression or knockdown/out) of the chromatin modifier | Other omics data in the same tissue/cell line |
| Targets of a TF in a certain perturbation | TF | Binding regions of the TF differential between two stages, before and after the perturbation | Transcriptome before and after the perturbation | Other omics data in either stage or differential signals between two stages |
| Targets of a chromatin modifier in a certain perturbation | Chromatin modifier | Binding regions of the chromatin modifier differential between two stages, before and after the perturbation | Transcriptome before and after the perturbation | Other omics data in either stage or differential signals between two stages |
| Targets regulated by a histone modification in a certain perturbation | Histone modification | Binding regions of the histone modification differential between two stages, before and after the perturbation | Transcriptome before and after the perturbation | Other omics data in either stage or differential signals between two stages |
| Targets regulated by DNA methylation in a certain perturbation | DNA methylation | Regions whose DNA methylation is differential between two stages, before and after the perturbation | Transcriptome before and after the perturbation | Other omics data in either stage or differential signals between two stages |

# 2 Curated Data

1. ChIP-X, long-range interaction data, open chromatin regions and histone modification data

Please download from <http://jjwanglab.org/chip-array-v2/media/ChIP-Array2_DataStatistics.xlsx>.

1. Experimental-validated enhancers

Please download from <http://jjwanglab.org/chip-array-v2/media/curated-enhancers.xlsx>.

# 3 mESC library

The mESC library is built based on the data collected from different sources (Supplementary Table S3).

Supplementary Table S3. Omics data for network construction

| Involved Factor | Experiment Type | Data Source | PubMed ID |
| --- | --- | --- | --- |
| Cdx2 | ChIP-seq | GSE16375 | 19796622 |
| Esrrb | ChIP-seq | GSE11431 | 18555785 |
| Jarid2 | ChIP-seq | GSE19365 | 20075857 |
| Jarid2 | ChIP-seq | GSE18776 | 20064375 |
| Kdm5a | ChIP-seq | GSE18776 | 20064375 |
| Klf4 | ChIP-seq | GSE11431 | 18555785 |
| Myc | ChIP-seq | GSE11431 | 18555785 |
| Mycn | ChIP-seq | GSE11431 | 18555785 |
| Nanog | ChIP-seq | GSE11431 | 18555785 |
| Nr5a2 | ChIP-seq | GSE19019 | 20096661 |
| Pou5f1 | ChIP-seq | GSE11431 | 18555785 |
| Pou5f1 | ChIP-seq | GSE22934 | 21477851 |
| Rest | ChIP-seq | GSE26680 | - |
| Rest | ChIP-seq | GSE27844 | 22297846 |
| Smad1 | ChIP-seq | GSE11431 | 18555785 |
| Sox2 | ChIP-seq | GSE11431 | 18555785 |
| Stat3 | ChIP-seq | GSE11431 | 18555785 |
| Suz12 | ChIP-seq | GSE11431 | 18555785 |
| Suz12 | ChIP-seq | GSE13084 | 18974828 |
| Suz12 | ChIP-seq | GSE19365 | 20075857 |
| Suz12 | ChIP-seq | GSE18776 | 20064375 |
| Tbx3 | ChIP-seq | GSE19219 | 20139965 |
| Tcfcp2l1 | ChIP-seq | GSE11431 | 18555785 |
| Nelfa | ChIP-seq | GSE20530 | 20434984 |
| Esrrb | Transcriptome under knockdown | GSE26520 | 23462645 |
| Jarid2 | Transcriptome under knockdown | GSE26520 | 23462645 |
| Klf4 | Transcriptome under knockdown | GSE26520 | 23462645 |
| Myc | Transcriptome under knockdown | GSE26520 | 23462645 |
| Mycn | Transcriptome under knockdown | GSE26520 | 23462645 |
| Nanog | Transcriptome under knockdown | GSE26520 | 23462645 |
| Nanog | Transcriptome under knockdown | GSE26520 | 23462645 |
| Nr5a2 | Transcriptome under knockdown | GSE26520 | 23462645 |
| Pou5f1 | Transcriptome under knockdown | GSE26520 | 23462645 |
| Pou5f1 | Transcriptome under knockdown | GSE26520 | 23462645 |
| Rest | Transcriptome under knockdown | GSE26520 | 23462645 |
| Sox2 | Transcriptome under knockdown | GSE26520 | 23462645 |
| Stat3 | Transcriptome under knockdown | GSE26520 | 23462645 |
| Suz12 | Transcriptome under knockdown | GSE26520 | 23462645 |
| Tbx3 | Transcriptome under knockdown | GSE26520 | 23462645 |
| Tcfcp2l1 | Transcriptome under knockdown | GSE26520 | 23462645 |
| Whsc2 | Transcriptome under knockdown | GSE26520 | 23462645 |
| Cdx2 | Transcriptome under overexpression | GSE16375 | 19796622 |
| Jarid2 | Transcriptome under overexpression | GSE31381 | 22355682 |
| Kdm5a | Transcriptome under overexpression | GSE31381 | 22355682 |
| Klf4 | Transcriptome under overexpression | GSE16375 | 19796622 |
| Myc | Transcriptome under overexpression | GSE16375 | 19796622 |
| Mycn | Transcriptome under overexpression | GSE16375 | 19796622 |
| Nanog | Transcriptome under overexpression | GSE16375 | 19796622 |
| Nr5a2 | Transcriptome under overexpression | GSE16375 | 19796622 |
| Pou5f1 | Transcriptome under overexpression | GSE16375 | 19796622 |
| Rest | Transcriptome under overexpression | GSE31381 | 22355682 |
| Smad1 | Transcriptome under overexpression | GSE16375 | 19796622 |
| Sox2 | Transcriptome under overexpression | GSE16375 | 19796622 |
| Stat3 | Transcriptome under overexpression | GSE16375 | 19796622 |
| Suz12 | Transcriptome under overexpression | GSE16375 | 19796622 |
| Tbx3 | Transcriptome under overexpression | GSE31381 | 22355682 |
| Tcfcp2l1 | Transcriptome under overexpression | GSE31381 | 22355682 |
| Whsc2 | Transcriptome under overexpression | GSE16375 | 19796622 |
| H3K4me3 | ChIP-seq | ENCODE | - |
| H3K4me1 | ChIP-seq | ENCODE | - |
| H3K27ac | ChIP-seq | ENCODE | - |
| H3K27me3 | ChIP-seq | ENCODE | - |
| H3K9me3 | ChIP-seq | ENCODE | - |
| Open chromatin region | DHS-seq | ENCODE | - |
| Long-range interaction | ChIA-PET | GSE44067 | 24213634 |

# 4 Comparison with ChIP-Array and other tools

1. Comparison with BETA and ChIP-Array

To evaluate the results of ChIP-Array 2 and related tools, we curated the experimentally validated targets of Sox2 and Pou5f1 in mESC from literature as the benchmark (Supplementary Table S4).

Supplementary Table S4. Experimentally validated targets of Sox2 and Pou5f1 in mESC

| Regulator | Target | Reference |
| --- | --- | --- |
| Sox2 | Fbxo15 | ([10](#_ENREF_10)) |
| Sox2 | Fgf4 | ([11](#_ENREF_11)) |
| Sox2 | Lefty1 | ([12](#_ENREF_12)) |
| Sox2 | Nanog | ([13](#_ENREF_13),[14](#_ENREF_14)) |
| Sox2 | Pou5f1 | ([12](#_ENREF_12),[14](#_ENREF_14),[15](#_ENREF_15)) |
| Sox2 | Sox2 | ([12](#_ENREF_12),[14](#_ENREF_14)) |
| Sox2 | Utf1 | ([12](#_ENREF_12)) |
| Sox2 | Xist | ([16](#_ENREF_16)) |
| Sox2 | Zscan10 | ([17](#_ENREF_17)) |
| Pou5f1 | Cdkn1a | ([18](#_ENREF_18)) |
| Pou5f1 | Cdx2 | ([19](#_ENREF_19)) |
| Pou5f1 | Ctcf | ([16](#_ENREF_16)) |
| Pou5f1 | Eed | ([20](#_ENREF_20)) |
| Pou5f1 | Fbxo15 | ([10](#_ENREF_10)) |
| Pou5f1 | Fgf4 | ([21](#_ENREF_21)) |
| Pou5f1 | Kdm3a | ([22](#_ENREF_22)) |
| Pou5f1 | Kdm4c | ([22](#_ENREF_22)) |
| Pou5f1 | Klf2 | ([23](#_ENREF_23)) |
| Pou5f1 | Lefty1 | ([12](#_ENREF_12)) |
| Pou5f1 | Nanog | ([13](#_ENREF_13),[14](#_ENREF_14),[24](#_ENREF_24)) |
| Pou5f1 | Pou5f1 | ([12](#_ENREF_12),[14](#_ENREF_14)) |
| Pou5f1 | Sox2 | ([12](#_ENREF_12),[14](#_ENREF_14)) |
| Pou5f1 | Tsix | ([16](#_ENREF_16)) |
| Pou5f1 | Utf1 | ([12](#_ENREF_12)) |
| Pou5f1 | Xist | ([16](#_ENREF_16)) |
| Pou5f1 | Yy1 | ([16](#_ENREF_16)) |
| Pou5f1 | Zfp57 | ([25](#_ENREF_25)) |
| Pou5f1 | Zscan10 | ([17](#_ENREF_17)) |

Since BETA do not detect indirect targets, we compared only the direct targets of Sox2 and Pou5f1 identified by BETA, ChIP-Array and ChIP-Array 2. For ChIP-Array 2, we incorporated long-range chromatin interaction data generated by ChIA-PET from GEO (GSE44067) ([26](#_ENREF_26)), which is not considered by either ChIP-Array or BETA. We used the default options for BETA except “-d”, “-c”, and “--df”. “-d” defines a distance from TSS that the peaks are takes into account. “-c” gives a cutoff to filter the true targets. “--df” specifies a statistical value cutoff that suggesting the differentially expressed genes being considered in construction of GRNs. These three options are 10000, 0.005, and 0.05, respectively, which are the same as the corresponding options in ChIP-Array 2.

As the results shown in Supplementary Table S5, the enrichment p value between ChIP-Array2 and the benchmark is much lower than that of BETA and ChIP-Array. Actually, ChIP-Array 2 and BETA used similar methods, however, BETA cannot incorporate long-range chromatin interaction data, which may help to find some targets regulated by distal elements (i.e. enhancers) with transcription factor involvement (Figure 2B in main text). ChIP-Array defines a target as a gene that is significantly differentially expressed and has binding events in its promoter region. It regards the targets equally, and does not give any rank on the targets. When the promoter region is set as the same as ChIP-Array 2, it detects too many targets. Even we shorten the region to -500~+100, it still gives more than 7000 targets, which results in a very high false positive rate. Differently, ChIP-Array 2 ranks the targets, allowing users to assign a cutoff to choose the more likely true targets.

Supplementary Table S5. Comparison of the results of different tools

| Tool | No. of targets detected | No. of targets hit the benchmark | Enrichment p-value with the benchmark^a^ |
| --- | --- | --- | --- |
| Factor: Sox2, benchmark size: 9 | | | |
| BETA | 384 | 1 | >0.05 |
| ChIP-Array | 7690 | 7 | 6.42E-4 |
| ChIP-Array 2 | 282 | 3 | 4.48E-5 |
| Factor: Pou5f1, benchmark size: 19 | | | |
| BETA | 1196 | 1 | >0.05 |
| ChIP-Array | 7184 | 12 | 7.36E-5 |
| ChIP-Array 2 | 271 | 6 | 5.86E-9 |

^a^: Enrichment is performed using hypergeometric test.

1. Comparison with other tools

Other tools require either additional data or a large number of samples to build the model for target detection, so we can’t compare them with our example data. For example, Maienschein-Cline, M., etc. ([29](#_ENREF_29)) proposed a method call “EMBER” to detect the targets. It needs time-serials expression data under TF perturbation to find the enriched expression change patterns around TF binding peaks, which costs more, so is not a common experiment design for TF target identification. Qin, *et al.* ([28](#_ENREF_28)) constructed GRNs using Least Absolute Shrinkage and Selection Operator (LASSO)-type models. They extended the models to integrate ChIP-X data. However, it needs a large number of samples to construct expression matrices for the calculation. PTHGRN ([27](#_ENREF_27)) incorporates protein-protein interaction (PPI) network and ChIP-seq data to identify the interplay between post-translational modification (PTM) and TFs. It also require a series of expression profiles to search tissue-specific PTM-TF interactions and their targets. Similar to ([28](#_ENREF_28)), Zhang, *et al*. ([30](#_ENREF_30)) discover functional modules considering multiple omics using a matrix factorization method, which apparently needs a large number of samples to solve the problem. Compared to these tools, ChIP-Array 2 provides a more flexible platform which can be applied to most of studies on gene regulation with different combination of omics data. Users can utilize the web server even though they have only one ChIP-seq or expression profiles from two experimental conditions. In addition, it emphasizes the tissue specificity by combining data from the same or, at least, similar tissue/condition. Users can use omics data matching the tissue/condition of their own data from our database to improve the target identification. Moreover, none of above tools analyze long-range chromatin interaction, open chromatin region and histone modification data simultaneously, although they are helpful to construct more comprehensive GRNs (Introduction in main text). And they do not detect indirect targets.

# Reference

1. Siepel, A., Bejerano, G., Pedersen, J.S., Hinrichs, A.S., Hou, M., Rosenbloom, K., Clawson, H., Spieth, J., Hillier, L.W., Richards, S. *et al.* (2005) Evolutionarily conserved elements in vertebrate, insect, worm, and yeast genomes. *Genome research*, **15**, 1034-1050.

2. Rosenbloom, K.R., Armstrong, J., Barber, G.P., Casper, J., Clawson, H., Diekhans, M., Dreszer, T.R., Fujita, P.A., Guruvadoo, L., Haeussler, M. *et al.* (2015) The UCSC Genome Browser database: 2015 update. *Nucleic acids research*, **43**, 81.

3. Zheng, Q., Ryvkin, P., Li, F., Dragomir, I., Valladares, O., Yang, J., Cao, K., Wang, L.-S.S. and Gregory, B.D. (2010) Genome-wide double-stranded RNA sequencing reveals the functional significance of base-paired RNAs in Arabidopsis. *PLoS genetics*, **6**.

4. Li, F., Zheng, Q., Vandivier, L.E., Willmann, M.R., Chen, Y. and Gregory, B.D. (2012) Regulatory impact of RNA secondary structure across the Arabidopsis transcriptome. *The Plant cell*, **24**, 4346-4359.

5. Korhonen, J., Martinmäki, P., Pizzi, C., Rastas, P. and Ukkonen, E. (2009) MOODS: fast search for position weight matrix matches in DNA sequences. *Bioinformatics (Oxford, England)*, **25**, 3181-3182.

6. Ouyang, Z., Zhou, Q. and Wong, W.H. (2009) ChIP-Seq of transcription factors predicts absolute and differential gene expression in embryonic stem cells. *Proceedings of the National Academy of Sciences of the United States of America*, **106**, 21521-21526.

7. Tang, Q., Chen, Y., Meyer, C., Geistlinger, T., Lupien, M., Wang, Q., Liu, T., Zhang, Y., Brown, M. and Liu, X.S. (2011) A comprehensive view of nuclear receptor cancer cistromes. *Cancer research*, **71**, 6940-6947.

8. Wang, S., Sun, H., Ma, J., Zang, C., Wang, C., Wang, J., Tang, Q., Meyer, C.A., Zhang, Y. and Liu, X.S. (2013) Target analysis by integration of transcriptome and ChIP-seq data with BETA. *Nature protocols*, **8**, 2502-2515.

9. Breitling, R., Armengaud, P., Amtmann, A. and Herzyk, P. (2004) Rank products: a simple, yet powerful, new method to detect differentially regulated genes in replicated microarray experiments. *FEBS letters*, **573**, 83-92.

10. Tokuzawa, Y., Kaiho, E., Maruyama, M., Takahashi, K., Mitsui, K., Maeda, M., Niwa, H. and Yamanaka, S. (2003) Fbx15 is a novel target of Oct3/4 but is dispensable for embryonic stem cell self-renewal and mouse development. *Molecular and cellular biology*, **23**, 2699-2708.

11. Luster, T.A. and Rizzino, A. (2003) Regulation of the FGF-4 gene by a complex distal enhancer that functions in part as an enhanceosome. *Gene*, **323**, 163-172.

12. Boer, B., Kopp, J., Mallanna, S., Desler, M., Chakravarthy, H., Wilder, P.J., Bernadt, C. and Rizzino, A. (2007) Elevating the levels of Sox2 in embryonal carcinoma cells and embryonic stem cells inhibits the expression of Sox2:Oct-3/4 target genes. *Nucleic Acids Res*, **35**, 1773-1786.

13. Rodda, D.J., Chew, J.L., Lim, L.H., Loh, Y.H., Wang, B., Ng, H.H. and Robson, P. (2005) Transcriptional regulation of nanog by OCT4 and SOX2. *The Journal of biological chemistry*, **280**, 24731-24737.

14. Chickarmane, V., Troein, C., Nuber, U.A., Sauro, H.M. and Peterson, C. (2006) Transcriptional dynamics of the embryonic stem cell switch. *PLoS computational biology*, **2**, e123.

15. Maruyama, M., Ichisaka, T., Nakagawa, M. and Yamanaka, S. (2005) Differential roles for Sox15 and Sox2 in transcriptional control in mouse embryonic stem cells. *The Journal of biological chemistry*, **280**, 24371-24379.

16. Donohoe, M.E., Silva, S.S., Pinter, S.F., Xu, N. and Lee, J.T. (2009) The pluripotency factor Oct4 interacts with Ctcf and also controls X-chromosome pairing and counting. *Nature*, **460**, 128-132.

17. Wang, Z.X., Teh, C.H., Kueh, J.L., Lufkin, T., Robson, P. and Stanton, L.W. (2007) Oct4 and Sox2 directly regulate expression of another pluripotency transcription factor, Zfp206, in embryonic stem cells. *The Journal of biological chemistry*, **282**, 12822-12830.

18. Lee, J., Go, Y., Kang, I., Han, Y.M. and Kim, J. (2010) Oct-4 controls cell-cycle progression of embryonic stem cells. *The Biochemical journal*, **426**, 171-181.

19. Niwa, H., Toyooka, Y., Shimosato, D., Strumpf, D., Takahashi, K., Yagi, R. and Rossant, J. (2005) Interaction between Oct3/4 and Cdx2 determines trophectoderm differentiation. *Cell*, **123**, 917-929.

20. Ura, H., Usuda, M., Kinoshita, K., Sun, C., Mori, K., Akagi, T., Matsuda, T., Koide, H. and Yokota, T. (2008) STAT3 and Oct-3/4 control histone modification through induction of Eed in embryonic stem cells. *The Journal of biological chemistry*, **283**, 9713-9723.

21. Schoorlemmer, J. and Kruijer, W. (1991) Octamer-dependent regulation of the kFGF gene in embryonal carcinoma and embryonic stem cells. *Mechanisms of development*, **36**, 75-86.

22. Loh, Y.H., Zhang, W., Chen, X., George, J. and Ng, H.H. (2007) Jmjd1a and Jmjd2c histone H3 Lys 9 demethylases regulate self-renewal in embryonic stem cells. *Genes & development*, **21**, 2545-2557.

23. Hall, J., Guo, G., Wray, J., Eyres, I., Nichols, J., Grotewold, L., Morfopoulou, S., Humphreys, P., Mansfield, W., Walker, R. *et al.* (2009) Oct4 and LIF/Stat3 additively induce Kruppel factors to sustain embryonic stem cell self-renewal. *Cell stem cell*, **5**, 597-609.

24. Pan, G., Li, J., Zhou, Y., Zheng, H. and Pei, D. (2006) A negative feedback loop of transcription factors that controls stem cell pluripotency and self-renewal. *FASEB journal : official publication of the Federation of American Societies for Experimental Biology*, **20**, 1730-1732.

25. Akagi, T., Usuda, M., Matsuda, T., Ko, M.S., Niwa, H., Asano, M., Koide, H. and Yokota, T. (2005) Identification of Zfp-57 as a downstream molecule of STAT3 and Oct-3/4 in embryonic stem cells. *Biochemical and biophysical research communications*, **331**, 23-30.

26. Zhang, Y., Wong, C.H., Birnbaum, R.Y., Li, G., Favaro, R., Ngan, C.Y., Lim, J., Tai, E., Poh, H.M., Wong, E. *et al.* (2013) Chromatin connectivity maps reveal dynamic promoter-enhancer long-range associations. *Nature*, **504**, 306-310.

27. Guan, D., Shao, J., Zhao, Z., Wang, P., Qin, J., Deng, Y., Boheler, K.R., Wang, J. and Yan, B. (2014) PTHGRN: unraveling post-translational hierarchical gene regulatory networks using PPI, ChIP-seq and gene expression data. *Nucleic Acids Res*, **42**, W130-136.

28. Qin, J., Hu, Y., Xu, F., Yalamanchili, H.K. and Wang, J. (2014) Inferring gene regulatory networks by integrating ChIP-seq/chip and transcriptome data via LASSO-type regularization methods. *Methods*, **67**, 294-303.

29. Maienschein-Cline, M., Zhou, J., White, K.P., Sciammas, R. and Dinner, A.R. (2012) Discovering transcription factor regulatory targets using gene expression and binding data. *Bioinformatics (Oxford, England)*, **28**, 206-213.

30. Zhang, S., Liu, C.C., Li, W., Shen, H., Laird, P.W. and Zhou, X.J. (2012) Discovery of multi-dimensional modules by integrative analysis of cancer genomic data. *Nucleic Acids Res*, **40**, 9379-9391.
